# Ontogenetic shifts in morphology and ecology of eastern Pacific white sharks revealed by computer vision

**DOI:** 10.1371/journal.pone.0348174

**Published:** 2026-05-20

**Authors:** Alexandra E. DiGiacomo, Samantha Andrzejaczek, Barbara A. Block

**Affiliations:** 1 Department of Biology, Stanford University Hopkins Marine Station, Pacific Grove, California, United States of America; 2 Department of Oceans, Stanford University Hopkins Marine Station, Pacific Grove, California, United States of America; James Cook University, AUSTRALIA

## Abstract

Body size is a fundamental property of animal physiology, growth, and maturation, yet field measurements remain difficult to acquire for large-bodied, highly mobile marine species such as white sharks (*Carcharodon carcharias)*. In this study, we integrate aerial and underwater imagery to obtain high-resolution morphometrics of eastern Pacific white sharks remotely in the Monterey Bay National Marine Sanctuary. We develop and validate a computational pipeline leveraging deep learning analysis of Unoccupied Aircraft System (UAS) imagery to extract shark total length and body condition, given by a span-length ratio. UAS-based morphometric data reveal that white sharks form size-structured aggregations aligned with oceanographic gradients, indicating that coastal areas within Monterey Bay function as key transitional zones along a continuum of ontogenetic habitat use on the central coast of California. Across individuals, extended girth-length scaling relationships indicate proportionally greater girth amongst eastern Pacific white sharks relative to other populations. This pattern is particularly pronounced in females, which exhibit progressively higher body condition with life stage, likely reflecting the energetic demands of reproduction or sex-specific foraging strategies. By linking UAS-derived morphometrics to ecological context, this approach enables a novel population-level investigation of ecological structure, body size, and morphological variation in a marine predator population.

## Introduction

Animal body size reveals critical demographic information and is important to animal physiology, kinematics, and population dynamics. Body size morphometrics provide insights into metabolic and growth rates, maturation, energetic stores, and developmental patterns across many vertebrate species [1]. In white sharks (*Carcharodon carcharias)*, body size serves as a proxy for determining ontogenetic stage and body mass via age-length and weight-length curves, which have been essential in recent assessments of white shark population size and dynamics [2–5]. Shark girth indicates body condition, where relative girth has been shown to reflect available lipid reserves across large shark species [6–8]. These relationships provide essential information for understanding white shark life history, underscoring the importance of accurate morphometric data.

White shark growth and morphometric relationships are well documented, though regional and demographic variation remain incompletely resolved. Individuals are broadly categorized into ontogenetic classes (juveniles, subadults, and adults) by length, with females attaining larger maximum sizes than males [9–11]. Body size is a primary driver of an ontogenetic diet shift from fish-based prey amongst juveniles to marine mammal predation in subadults and adults [12,13], underscoring the ecological importance of resolving size-at-stage across life history. Direct measurements of sharks captured as bycatch in fisheries post-mortem [11,14] have provided data for computing age- and mass-length relationships as well as the scaling of more complex morphological features like girth [7,15], caudal fin, [16] and pectoral fin [15] morphometrics. However, published relationships have various approaches to incorporating girth, sex, and ontogenetic stage, and are often applied across regional populations despite evidence of geographical variation [11,14,17,18]. Such variability hinders the accurate estimation of growth trajectories, size-at-stage, and ontogenetic transitions.

Measurements of white shark body size at scale have become increasingly relevant in the eastern Pacific population, where shifting distributions have concentrated multiple age classes of white sharks in the Monterey Bay National Marine Sanctuary (MBNMS). The MBNMS extends from north of San Francisco to south of the Big Sur region, encompassing over 400 km of California coastline [19]. While subadult and adult white sharks have been known to seasonally concentrate at pinniped haul-out sites in this area before performing annual offshore migrations [20,21], juvenile sharks were historically observed to the south in the warmer, more protected waters of the Southern California Bight while foraging on bony fish and small elasmobranchs [21–23]. A recent rise in the observations of juvenile white sharks along the central California coast began after a marine heat wave in 2015, where warm Pacific subtropical gyre waters impacted this area [24]. Concurrently, fisheries regulations that had closed California gill netting operations began to prompt recovery of similarly sized coastal megafauna like harbor porpoises [25]. Over the past decade, juvenile and subadult white sharks have been increasingly aggregating in protected habitats inside Monterey Bay as evidenced by local reports, citizen scientist data, and the rise of shark bites on sea otters and harbor seals [26–28]. Characterizing morphometrics across age classes in this population is essential for resolving a shifting ecological landscape, yet doing so presents substantial practical challenges.

Acquiring field measurements from live white sharks can be logistically difficult owing to their large body sizes, high mobility, and pelagic lifestyles. Direct measurements of mature individuals via catch-and-release requires specialized equipment and complex animal handling procedures [9], biasing recent measurements toward smaller individuals in the juvenile age class [7,9,15]. Remote measurement methods using underwater imaging, like parallel laser photogrammetry [29,30] and stereo-video rigs [31] have been explored, but require close proximity to the animal. Due to these constraints, researchers typically estimate white shark total length visually. Previous verifications of visual length estimate accuracy, however, reveal substantial variability [31] and fail to resolve more complex morphometric parameters, like girth, motivating reproducible and accurate methods of measuring shark morphometrics when performing demographic studies.

With the recent rise of UAS, or drones, for scientific research, researchers have explored the ability of aerial photogrammetry to compute free-swimming morphometric estimates in similarly logistically challenging animals [32–35]. Primarily amongst marine mammals, researchers quantify body widths, or spans, and have developed metrics of body condition to track animal health and foraging success [33]. While these methods can be difficult to apply across pelagic species that spend a large proportion of their lives undetectable from the surface at depth, UAS can present a useful data collection platform for species that frequent surface waters, like white sharks [36]. Increasing aerial observations have been complemented by advances in computer vision analysis techniques, which have been used to detect and localize white sharks in surveys [37,38] and remotely extract kinematic parameters from focal follows [39]. Computer vision models have been successfully employed to extract animal morphometrics in other species [40–44], but these techniques have not been comprehensively applied and validated for white sharks. Some studies have begun to measure white sharks using photogrammetry, but measurements often lack validation, contain error from uncertainty in UAS altitude and animal depth [45], and cannot be reliably linked to individual identification from aerial data alone. Collectively, these limitations have constrained the ability to compare white shark field measurements across life stages and ecological contexts.

The co-occurrence of recently formed juvenile hotspots with well-studied adult and subadult white shark aggregations in the MBNMS presents a unique system in which to directly compare morphometrics across ontogeny using a standardized approach. In this study, computer vision techniques are employed to extract morphometric data from aerial imagery of white sharks across multiple aggregation sites in the eastern Pacific. The goals of this study are to (1) develop and validate an image-based approach to extracting white shark morphometrics and (2) apply this approach to investigate how spatial habitat partitioning, morphometric scaling relationships, and body condition change across ontogeny.

## Materials and methods

Aerial images of white sharks were collected at three seasonal aggregations in the MBNMS from 2022-2025. Inside Monterey Bay, which spans from Santa Cruz to the north and Monterey Peninsula to the south, white sharks were observed in two shallow sandy beach habitats in the coastal waters off New Brighton State Beach and Marina State Beach. These areas have recently been noted as white shark aggregation areas for juvenile white sharks in summer to fall months [27,39]. New Brighton State Beach, situated in the northern elbow of Monterey Bay, is protected from wind stress and maintains warmer water temperatures in summer and fall relative to the surrounding area, while Marina State Beach occupies a slightly more exposed region of the bay with cooler water temperatures [46,47]. The third focal aggregation, Año Nuevo, is located off Año Nuevo Island, just outside of Monterey Bay along the northern coastline. These waters experience the coldest water temperatures and are characterized by a rocky seafloor, reefs, and deeper channels [48]. This location has served as a long-term study site for subadult and adult white sharks in fall to winter months, as sharks forage on marine mammal colonies present on the island [20,21,49].

White sharks were observed with a DJI Phantom 4 Advanced quadcopter, equipped with a 1-inch 20-megapixel CMOS sensor. The drone was deployed from a small vessel and conducted in compliance with Federal Aviation Administration 14 CFR Part 107 regulations and MBNMS permits (Permit MULTI-2023–005). At nearshore sites inside Monterey Bay (New Brighton, Marina), individuals were located by UAS and the vessel was directed to approach the sharks. At Año Nuevo, individuals were attracted to the surface using a decoy and olfactory cues from a permitted release of marine mammal bait (MBNMS Permit MULTI-2023–005). When a shark was located, still images (n = 5–10) were collected for body measurements at 10-70m altitude and a nadir gimbal angle. At the end of each UAS flight, still images (n = 3–5) of a calibration object of known length deployed at the surface were collected to calibrate UAS-based altitude readings.

Once measurements were obtained, the focal white shark was approached by a small research vessel for underwater identification using a GoPro (GoPro, Inc., San Mateo, CA, USA) attached to an aluminum extendable pole. Individuals were identified via underwater footage of the dorsal fin, which is an established method for re-identifying animals in this population [2,5]. Video data was also used to determine sex, which was classified by the presence (M) or absence (F) of claspers on the ventral side of the animal. UAS data were paired with individual identification and sex information. Visual, or ‘by-eye’, estimations of animal size were recorded as the average of three experienced researchers’ estimates, recorded to the nearest 0.5 ft. Water temperature was recorded at the beginning of each field day using a calibrated Hanna Instruments handheld thermistor at the surface or a calibrated RBR Ruskin Conductivity Temperature Depth (CTD) device. All methods were performed in accordance with relevant guidelines and regulations including MBNMS Permit MULTI-2023–005 and all experimental protocols were approved under Stanford University Institutional Animal Care protocol 10765.

### Model development and validation

The UAS imagery dataset was curated through a two-stage process involving visual review for quality control and structured splitting for model development. Initially, UAS images were reviewed to ensure that both shark pectoral fins were discernible and the shark’s body was largely unobstructed. Then, a subset of images received annotations of the shark body, excluding pectoral fins, traced using the polygon tool in Computer Vision Annotation Tool (CVAT) [50] to isolate the mask, or pixel-level body region. Annotated images were then split into train, validation, and test subsets (~70%/15%/15%). Images from a single drone flight on a given day were assigned to the same split to prevent model overfitting, ensuring that sequential images of the same individual under similar environmental conditions did not appear across sets. The training and validation sets were used during model development to compare model configurations, while the test set was held out and used only once to evaluate final model performance. All images were annotated with shark body center coordinates in annotation platform CVAT [50] to support image cropping procedures used in later steps.

To evaluate the model’s performance in terms of morphometrics, traits were digitally measured to provide ‘ground truth’ data using image processing software package Fiji [51]. Shark total length (TL) was manually traced using the multi-segmented line tool along the back of the individual, with points on the tail tip, caudal peduncle, second dorsal fin, dorsal fin, and nose to address body flexion. This was complementary to the traditional visual, ‘by-eye’ TL estimations performed on the boat. In addition, body span metrics Lateral Span (LS), Frontal Span (FS), and Proximal Span (PS) were digitally measured using the straight line tool at anatomically relevant positions. Spans were measured as flat, non-curved body widths at the anterior edge of the pectoral fins (LS) and the anterior (FS) and posterior (PS) insertion points of the dorsal fin [52].

Five model configurations were evaluated that varied image cropping and input resolution, following common practices for computer vision models optimized for fixed-size image inputs [53].The *baseline* model used full original RGB UAS images (1080x1920-3648x5472) resized to 224 × 224 pixels, while the remaining models cropped image regions around manually annotated shark body center coordinates. The *Sharkcrop_medium* and *Sharkcrop_large* models implemented fixed-size crops of 448 × 448 and 896 × 896 pixels, respectively, resized to 224 × 224 pixels. For the *Sharkcrop_altitude* model, we implemented a dynamic cropping strategy, computing the minimum crop size necessary to encompass a maximum expected shark TL of 550 cm [5], based on UAS altitude and image width, rounded up to the nearest multiple of 112 pixels and then resized to 224 × 224. Finally, the *Sharkcrop_altitude_highres* model employed the same dynamic cropping method of *Sharkcrop_altitude* but retained a higher input resolution of 448 × 448 pixels.

To isolate the shark body mask, or region corresponding to the shark body, a U-Net segmentation model was trained from the segmentation_models_pytorch (SMP) library. This model uses a ResNet-34 encoder initialized with ImageNet pre-trained weights. This approach, known as transfer learning, allows the model to build on patterns learned from a large image database (ImageNet) to increase performance on a smaller, task-specific dataset common in ecology [53,54]. Model inputs were RGB imagery and predictions were binary segmentation masks representing the shark body excluding pectoral fins. All models were trained using AdamW optimization with a learning rate of 1e-4 and binary cross-entropy with logits loss. Models were trained for 100 epochs with a batch size of two images and validation was performed after every epoch (see code repository). The best performing model within each run was selected by comparing the validation performance in terms of Intersection Over Union (IoU). IoU, a common metric in segmentation analyses, is defined as the ratio of the overlapping area between the model-predicted mask and the annotated ground truth mask to their union area [55]. Masks were post-processed to isolate the shark body and remove artifacts while retaining morphological features of interest. The shark body was identified as the largest connected component using contour detection and retained as the main mask. Morphological dilation was used on the main mask to create a 10-pixel buffer zone, outside of which artifacts were removed. Adjacent anatomical features like the caudal fin that were disconnected but within the 10px buffer zone were reconnected to the main mask by a 1px width line.

**Fig 1:**
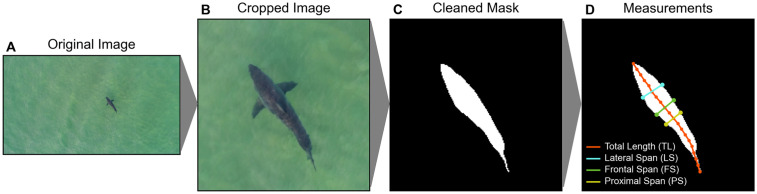
Schematic representation of UAS image segmentation and morphometric extraction. Methods schematic demonstrates progression from **(A)** original full-resolution imagery to **(B)** cropped 224x224 model inputs, **(C)** segmentation masks post-processed with cleaning functionalities, and **(D)** measurement extraction from mask properties. Mask measurements in pixels were then converted to true measurements via photogrammetry equations and calibrations to extract shark total length, body span (calculated as the average of Lateral Span, Frontal Span, and Proximal Span), and body span ratio (BSR).

Morphometric estimates of shark TL and body spans were derived from cleaned masks of the shark body (Fig 1). Shark TL was computed using the medial center line of the mask, computed from the skeletonize() function in scikit-image (Fig 1D). Skeletonized lines were pruned of forks and iteratively extended to reach the anterior and posterior mask edges using direction vectors from nearby skeleton points. To reduce noise and smooth the midline, we down sampled the extended skeletonized line to 20 evenly spaced points and computed TL as the sum of Euclidean distances between consecutive points.

Shark body condition was derived from cross-sectional widths, or spans, perpendicular to the resampled medial line (Fig 1D). Body spans were computed at 5%TL intervals, computed by extending lines outwards in both perpendicular directions until intersecting the mask boundary. To ensure consistent orientation, the head was identified by comparing the mean width of the first three spans (5%, 10%, 15% TL positions) versus the last three spans (85%, 90%, 95% TL positions) along the body. The larger value was designated as the head, and measurement orientation was set from the nose (0% TL position) to the caudal fin tip (100% TL position). Visual inspection was used to map span metrics LS, FS, and PS to computed percentiles, assigning them to the 25th (LS), 40th (FS), and 50th (PS) %TL positions (Fig 1D). The body condition metric Body Span Ratio (BSR) was computed as the average of these three body span metrics divided by TL to capture multiple body locations around the mid-body region that may show condition signals [52].

Model performance was evaluated on the validation dataset by computing the coefficient of determination (R²) between TL estimates from model-generated masks and ImageJ-derived ground truth measurements. We reported performance for all model configurations in terms of measurement R^2^ and IoU associated with the selected training epoch. We compared performance across configurations and selected the final model with the highest validation set R^2^ values.

The final model was used to generate mask predictions on the entire dataset, including data used in model development (train, evaluation, test) and an additional deployment dataset consisting of unlabeled data collected during the same time period. Mask predictions and the associated morphometrics (TL, LS, FS, PS) computed in post-processing were visualized for each sample and visually inspected for inaccuracies. Predictions were visually reviewed, and those in which the mask did not reflect the contour of the body or the placement of morphometric markers were inaccurate were dropped from further analysis.

To compute true measurements, we converted pixel-based body measurements to absolute units using calibrated photogrammetric equations. Pixel-based measurements were converted to centimeters using the ground sampling distance (GSD, cm/pixel), calculated as:


GSD = (H * Sw * 100) / (Fl * ImW) (1)

Where the H is the height of the UAS camera above the measurement object of interest (m), sensor width (Sw) and focal length (Fl) are intrinsic camera properties (mm), and image width (ImW) is the width of the image (pixels) [56]. Multiplying object length in pixels by the GSD produced true measurements (cm). For all images, barometric altitude, sensor width, focal length, image width, and gimbal angle were scraped from the image Exchangeable Image File (EXIF). We ensured images were nadir-oriented and free from distortion due to camera angle by excluding both calibration and shark images captured at gimbal angles shallower than 80°.

Calibration images were used to compute a flight-based altitude offset between barometric and true altitude. Objects of known size contained in calibration imagery were measured by manually annotating object end points in CVAT and computing the Euclidean distance between endpoints. The known length of the calibration object was used to back-calculate true aircraft altitude (see Eqn (1)) and barometer-recorded altitude was scraped directly from the image EXIF data. The offset between barometer-recorded and true altitude was calculated per-image and averaged over each UAS flight to provide a flight-based altitude correction.

In each shark image, height (H) was computed as the barometric altitude adjusted by the calibration-derived offset and estimated swimming depth. The flight-based altitude correction was used as a fixed value by which to adjust shark image altitudes in the corresponding flight. Additionally, a fixed addition of 1.0m was added to all shark image altitudes to account for swimming depth, or the estimated depth of the shark’s center of mass in the water column [45]. Therefore, for shark measurements, height (H) for GSD calculations was computed as:


$$H\;=\;({offset}_{flight}\;+{depth}_{body}+\;{alt}_{bar})$$ (2)

Where flight offset (offset_flight_) is the within-flight mean offset based on calibration imagery, swimming depth (depth_body_) is the estimated sub-surface depth of the shark body, and barometric altitude (alt_bar_) is the UAS-recorded altitude reading. Shark measurements were then converted to true measurements by multiplying by the GSD.

### Error analysis

To evaluate sources of measurement error in our approach, uncertainty in UAS-recorded barometric altitude, shark swimming depth, and segmentation mask predictions were all considered. To investigate potential variability in barometric altitude, which is anticipated to be the dominant source of uncertainty [57], we explored the offset between barometer-recorded altitude and true altitude in calibration imagery. To quantify systematic error in altitude across flights, the mean altitude offset was computed for each flight and the overall distribution was characterized in terms of mean±standard deviation (SD). To evaluate error within a flight, the residual altitude offset for individual images was computed by subtracting the per-flight mean offset. The SD of the resultant distribution was computed, which captures altitude error across images during a single flight, removing systematic biases. The within- to the across- flight SD was then compared and expressed as a variance ratio. This ratio indicated whether the altitude error was driven predominantly by systematic biases in the aircraft sensor or barometric variability within flights. We also addressed the possibility of barometric drift within a flight, as calibration imagery was typically recorded in a short time span at the end of each flight. A subset of flights was analyzed in which calibration images were acquired intermittently throughout the flight (~5–15 minutes apart) and the within-flight SD of the residual altitude offsets was calculated and compared to across-flight variability using a variance ratio.

Using our within-flight correction approach, the contributions of remaining photogrammetric uncertainty were evaluated across altitudes. Swimming depth was approximated to an uncertainty of ±0.25m, encompassing shark swimming depths between 0.75-1.25m, and combined with within-flight altitude error in quadrature to represent total photogrammetric error affecting effective height (H). The resulting uncertainty in H (±1SD) was propagated through the GSD equation (Eqn (1)) to compute the corresponding %TL error for each shark image. The inflection point, or area of maximum curvature, of the resulting error-altitude curve was identified and used to define a conservative altitude threshold by which to remove samples with high uncertainty.

Combined error from barometric altitude, shark swimming depth, and mask predictions was then used to characterize error across our dataset. Mask measurement error was computed as the standard deviation of the distribution of differences between mask (model prediction) and ground truth (ImageJ) measurements detailed in previous sections. We combined photogrammetric error and mask measurement error in quadrature to produce a singular estimate of measurement error. After employing the altitude threshold, the error distribution was characterized across the shark imagery dataset as both a proportion of TL (%) and in true measurements (cm).

To evaluate the observed precision of length measurements under varying imaging conditions, we used images of re-sighted individuals within a sampling day. We computed the coefficient of variation (CV) in TL estimates for individuals observed across at least two flights. We compared CV within a flight, reflecting similar altitude and environmental conditions, and across separate flights on the same day, with more variation in conditions such as sun angle, water coloration, and surface texture.

Finally, we compared visual ‘by-eye’ estimations of TL with validated UAS-based measurements. Visual estimates, which have historically provided important age class information in this population, were recorded in binned feet (0.5 ft increments) and converted to centimeters. We used Pearson’s correlation coefficient to quantify the linear association between mask and visual measurements and analyzed the resultant residuals (visual-UAS). Summary statistics. including mean residual and standard deviation of residuals, were reported to quantify the accuracy of visual estimations.

### Ecological application

After establishing and validating this novel measurement approach, the methodology was applied to examine ecological patterns across white sharks in the MBNMS. Specifically, the measured morphometric data was utilized to analyze (a) spatial habitat partitioning, (b) morphometric relationships, and (c) body condition across demographic groups. Body morphometrics (TL, LS, FS, PS, BSR) were averaged across all images of a given individual within a sampling day. Although some individuals were re-sighted over multiple days, we treated each day-specific observation of an individual (‘day-individual’) as a distinct measurement given the potential for growth in TL and change in body condition over time. To evaluate bias introduced from repeated sampling of an individual over multiple field days, analyses were also run using measurements averaged across all sightings per biological individual. Comparing morphometrics across groups, all relevant distributions were tested for normality (Shapiro-Wilk test) and homogeneity of variances (Levene’s test). Based on those diagnostics, the appropriate parametric or nonparametric statistical tests were implemented as described below.

(a) **Spatial Habitat Partitioning**

To compare the size structure across aggregations, the distribution of TL was compared across the three field sites (New Brighton (NB), Marina (MB), Año Nuevo (AN)). A Kruskal-Wallis H-test was implemented to examine site differences in average TL, followed by pairwise analyses using Mann-Whitney U-tests. Additionally, as females reach higher terminal body sizes and maturity cutoffs differ across sex [10,11], analyses were run separately for both males and females to check for effects of sex ratios that may confound site differences. Based on TL and sex, we also described the sites in terms of age class composition, where individuals are classified as juveniles (<300 cm TL), subadults (M: 300–360 cm TL, F:300–450 cm TL), or adults (M: > 360 cm TL, F: > 450 cm TL) [10,11]. To characterize the thermal regimes associated with each aggregation site, distributions of site temperature recorded on each sampling day were produced.

(b) **Morphometric relationships**

To evaluate the appropriate regions that reflect body condition signals, we examined all body widths along the shark center line computed at 5%TL intervals from nose to caudal fin tip. For each percentile interval, the raw span (cm) was computed as well as the span-length ratio (span/TL) across all sharks in the dataset. Mean span and standard deviation at each interval was used to identify the region of maximal body span. Body span and span-length ratio were averaged by site at each interval to examine body regions of high variability. These data were compared to the spans used to compute BSR (LS: 25%TL, FS: 40%TL, PS: 50%TL) to validate the relevance of commonly utilized anatomical measurement positions.

Span-length scaling relationships were evaluated for differences across measurement positions and demographic groups. Linear relationships were evaluated between TL and each of three individual span metrics (LS, FS, PS). We then analyzed the relationship between TL and average body span (mean(LS, FS, PS)) separately for males and females. These relationships were characterized using Pearson's correlation coefficient (r), corresponding p-values, and linear model coefficients. Both sex- and age class-based differences in span-length scaling relationships were assessed using generalized least squares (GLS) regression with an interaction term.

Body span was converted to estimated girth to compare our data with published girth-length scaling relationships derived from direct measurements of white sharks post-mortem. To do this, we isolated FS, a span measurement just along the leading edge of the first dorsal fin [52], which was approximated as the 40%TL body position. We converted FS to a circular girth by approximating the cross-section in this body area as a circle. This has been done in previous studies that assume the white shark form to be a prolate spheroid shape and the cross sections perpendicular to the long axis are circular [14,58]. Under this assumption, we converted UAS-derived FS (diameter) to estimated girth (circumference) and compared girth-length scaling to those found in juvenile eastern Pacific white sharks [7], Australian white sharks [15], and globally [14].

(c) **Body condition**

To contextualize our body condition metric (BSR) with published metrics, body condition ratios were computed using previously employed methodologies for whales and other large predatory sharks. Body Area Index (BAI), a scale-invariant metric used to compute body condition from UAS imagery of blue and gray whales, was estimated in accordance with Burnett et al. (2018). Span Condition Analysis (SCA), a directly-measured index validated to predict energy stores in tiger sharks [8], nurse sharks [59], and epaulette sharks [60], was computed in accordance with Irschick & Hammerschlag (2014). To approximate direct measurements, TL was converted to fork length (FL) using published relationships [7], FS, LS, and PS were converted to circular girth, and caudal keel circumference was approximated as a quarter of the PS girth. The alignment of both BAI and ASC was evaluated with our girth-length ratio (BSR) using Pearson’s correlation.

BSR was compared across spatial aggregations and demographic groups. We first examined the scaling relationship between BSR and TL to assess potential size effects. Relationships for females and males were evaluated separately using Pearson's correlation. We then examined differences in BSR across spatial aggregations using a one-way ANOVA of BSR across sites followed by Tukey’s HSD post-hoc tests to identify pairwise differences. Within each site, we ran independent t-tests to compare BSR differences by sex. We assessed ontogenetic variation, defined by sex-specific TL cutoffs, by analyzing BSR across age classes (juvenile, adult, subadult) with a one-way ANOVA and post-hoc pairwise comparisons.

## Results

In this study, a total of 1974 measurable white shark images and 938 calibration images were collected from June 2022-October 2025. These data were collected during 63 field sampling days and 258 unique flights across 180 identified sharks classified as day-individuals. The resultant imagery encompassed a range of camera altitudes (mean: 25.9m, range: 2.2-70m), image resolutions (1080x1920 - 3648x5472 pixels), and a variety of oceanic conditions such as water clarity, coloration, surface texture, and glare.

### Model development and validation

A subset of imagery for model development was curated and split across train/validation/test sets (%: 70/15/15; n:888/171/183 images). The model development subset encompassed 142 identified day-individuals and each split contained representation from all three field sites. Train and validation sets consisted of flight-date groups randomly assigned from 2023 and 2024 data, while the test set consisted of flight-date groups randomly assigned from 2022 and 2024. Unseen data from 2022 was included in the test set to evaluate model generalizability to unseen field conditions and individuals.

After mask cleaning and post-processing, all model configurations demonstrated relatively high performance in terms of evaluation metric R^2^ and IoU. The final model, *Sharkcrop_altitude*, demonstrated the highest performance, with close alignment between mask and ground truth TL values (validation R^2^: 0.996). The final model generalized well to new data, with high performance on the test set representing new, previously unseen, flights and including a new year of sampling data (test R^2^: 0.976) (Table 1).

**Table 1:** Model performance across configurations, ranked by highest performance.

model	validation R^2^	validation IOU	test R^2^
Sharkcrop_altitude	0.996	0.955	0.976
Sharkcrop_highres_altitude	0.972	0.959	—
Sharkcrop_large	0.823	0.953	—
Baseline	0.780	0.904	—
Sharkcrop_medium	0.369	0.957	—

Model performance was evaluated as the R^2^ between mask-derived total length (TL) and ground truth TL on the validation set (n = 171 images), which was used to select the final model (Sharkcrop_altitude). Test set performance (n = 183) was evaluated only for the final model.

The post-processing steps of mask cleaning and skeleton extension contributed to the observed high model performance. Testing contributions on the final model (*Sharkcrop_altitude*), post-processing enabled small improvements in both the validation set (ΔR^2^=+0.002) and test set (ΔR^2^=+0.003) performance. Skeleton pruning and extension yielded slightly larger improvements in the validation set (ΔR^2^=+0.012) and test set (ΔR^2^=+0.020).

Mask predictions were generated for 1358 images with paired underwater identification and measurement calibration data for 168 day-individuals. After dropping erroneous masks via visual review (n: 129), 1229 images representing 165 unique day-individuals remained.

### Error analysis

Altitude accuracy derived from calibration imagery demonstrated higher across-flight than within-flight variability (S1 Fig). Across 236 unique flights with paired calibration data, altitudes were underestimated by an average of 2.5m with high deviation (mean±SD: 2.46 ± 2.38m), indicating that employing a global fixed offset would still leave samples with high possible error (S1A Fig). Within flights, mean-centered altitude data showed variability of less than 1m (SD: ± 0.60m), demonstrating more consistent barometric information within a flight (S1B Fig). The variance ratio of within- to across-flight altitude offsets was 0.06, indicating that variability was strongly driven by across-flight differences. Checking for within-flight barometric drift, a subset of six flights were analyzed that had calibration imagery taken from 6.6-18.3 minutes apart (mean±SD: 11.8 ± 4.39 min). The variance ratio for this subset was 0.33, also indicating a strong effect of across-flight variation.

Combined sources of uncertainty from both photogrammetry and mask predictions provided a comprehensive understanding of the possible variability in estimates of shark length. Photogrammetric error (%TL) attributable to uncertainty in effective height, barometric, and swimming depth uncertainty demonstrated a strong inverse relationship with altitude, decreasing hyperbolically as altitude increased. The curve exhibited an inflection point at approximately 5m, beyond which error decreased more gradually (S1C Fig). A conservatively defined altitude threshold of >10m was assigned for all subsequent analyses to ensure low measurement uncertainty, reducing the sample size to 1076 images across 163 day-individuals. The relative contributions of error sources to total uncertainty were 49.5% barometric altitude, 8.6% swimming depth, and 41.9% mask measurements. Combining all error sources, expected %TL error and TL error were centered around ±4% (mean±SD: 3.90 ± 1.04%) and ±12 cm (mean±SD: 11.88 ± 3.16 cm), respectively.

To validate these modeled uncertainties with empirical observations, repeated measurements of individual sharks (day-individuals) within the same field day were analyzed. Re-observations of 36 individuals that were observed in two or more flights on the same sampling day demonstrated low variability and high measurement precision. Both within- and across-flight CV values were centered around 4–5% (within-flight mean±sd: 4.82 ± 2.88, across-flight mean±sd: 4.44 ± 3.36). Expressed as CV, this indicates that repeated TL estimates for the same shark varied on average by 4–5% of the mean estimated TL.

Visual estimations of TL, which have historically provided age class and size data in this population, demonstrated consistent underestimations of TL. Visual estimations were correlated with UAS-based measurements (n = 89, r = 0.776, p < 0.001), but systematically underestimated shark size. Visual estimation residuals were centered around underestimations of −29 cm with high variation (mean±SD: −28.79 ± 44.78 cm).

### Ecological application

Morphometric data for individual white sharks were computed for 163 day-individuals across 1076 images after implementing calibration, visual mask review, and altitude thresholding. Image-based measurements were averaged across each day-individual (mean±sd: 6.60 ± 4.51 images per day-individual). Morphometrics summarized per biological individual (n = 134) did not change statistical relationships and effect directions (S2 Fig).

(a) **Spatial Habitat Partitioning**

**Fig 2:**
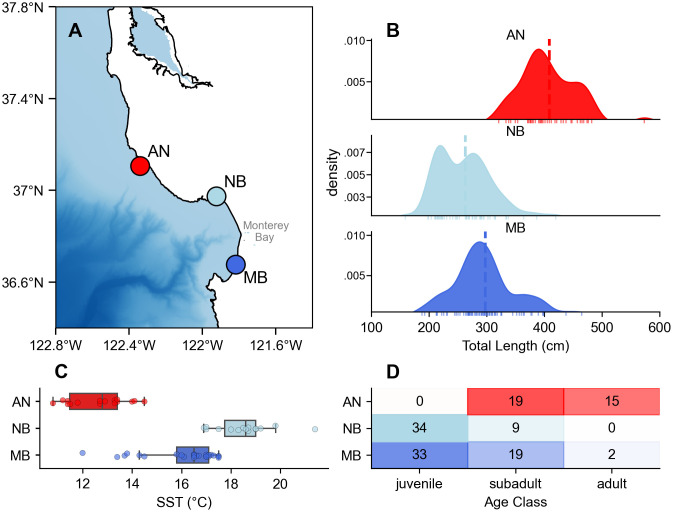
Site-level comparisons of shark size structure and thermal regimes. **(A)** Map of Monterey Bay National Marine Sanctuary (MBNMS), denoting sites Año Nuevo (AN), New Brighton (NB), and Marina (MB). **(B)** Distributions of total length across the three different sites using all individuals (n = 163). **(C)** Sea Surface Temperature (SST) regimes during sampling periods at each of the three sites. **(D)** Age class representation by site, determined by sex-dependent length cutoffs (n = 131), where cell numbers indicate (n).

Substantial differences were identified in mean TL distributions across the three aggregation sites in the MBNMS (Kruskal–Wallis H-test: p < 0.001; Fig 2B), with pairwise tests revealing significant differences between all site pairs (all corrected p < 0.001). TL distributions increased from NB (mean±sd: 262.7 ± 51.02 cm), to MB (297.4 ± 55.61 cm), to AN (408.4 ± 50.65 cm) (Fig 2B). These results were consistent when testing separately for males and females (H-test p < 0.001 (M, F), U-test p < 0.001 all pairs (M, F)), indicating that TL patterns across sites were not driven by differing sex ratios. Size class compositions reinforced the findings of TL. Using sex-length cutoffs to establish age class, the sites within Monterey Bay (NB, MB) were primarily dominated by juveniles and subadults, whereas AN exclusively hosted subadults and adults (Fig 2D).

These length and age class distributions were broadly aligned with thermal regimes during the corresponding sampling periods. AN had the coolest water temperatures during surveys (mean:12.6 ± 1.6°C) and the largest sharks, MB had intermediary temperatures (mean:16.0 ± 1.5°C) and intermediately sized sharks, and NB had the warmest water temperatures (mean:18.5 ± 1.2°C) and the smallest sharks (Fig 2C).

(b) **Morphometric relationships**

**Fig 3:**
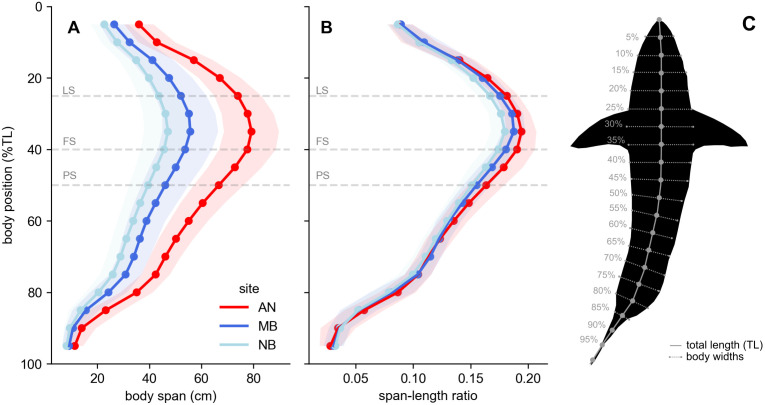
Whole-body span measurements along the shark center line. **(A)** Body span measurements derived at 5%TL intervals along the shark body center line colored by site and the corresponding **(B)** body span standardized to total length (TL). Bolded points represent site averages and shaded regions represent ±1SD. Dashed lines indicate the body position of measured Lateral Span (LS), Frontal Span (FS), and Proximal Span (PS). Site acronyms represent Año Nuevo (AN), New Brighton (NB), and Marina (MB). **(C)** Schematic example demonstrating center line and corresponding span collected at 5%TL intervals.

Examining trends in body spans along the shark center line, we observed the strongest signal of condition around the mid-body region (Fig 3), at similar positions to manual measurement positions used in previous studies [52]. The largest body spans were observed from the 25–45%TL positions, where the average span across all sharks was 53.0-58.4 cm and span-length ratio was 0.169-0.187 (Fig 3A, 3B). The widest overall span was the 35%TL position (mean span: 58.44 ± 15.9 cm, mean span-length ratio: 0.187 ± 0.012), in between the LS (25%TL) and FS (40%TL) biometric marker positions. Examining body spans across sites, we observed distinct span measurements owing to differences in body size but similar span-length ratios across the body. Differences in span-length ratios appeared to be most pronounced in the 25–50%TL region (Fig 3B), a region that encompasses our three utilized span metrics (LS, FS, PS).

Morphometric span variables (FS, LS, and PS) scaled linearly with TL (Table 2). All span metrics exhibited strong linear relationships with TL (Pearson’s r = 0.942-0.967). FS demonstrated the largest span values, while LS and PS demonstrated progressively smaller values consistent with anatomical expectations (Table 2). Analyzing sex-based differences, males demonstrated a lower slope coefficient (a = 0.17) than females (a = 0.19) in span-length relationships. This difference was confirmed by a significant interaction term between sex and TL in the GLS regression (p < 0.01) indicating a difference in span-length scaling across sex (Table 2, S3 Fig). No significant differences in slope or intercept were observed across age classes.

**Table 2:** Scaling relationships among span metrics and Total Length (TL).

Eqn	a	B	r	n	units
FS = a*TL + B	0.197	−4.84	0.958	163	cm
LS = a*TL + B	0.184	−2.92	0.967	163	cm
PS = a*TL + B	0.166	−2.93	0.942	163	cm
BS = a*TL + B (F)	0.193	−6.60	0.969	85	cm
BS = a*TL + B (M)	0.166	1.47	0.974	46	cm
BS = a*TL + B (juv)	0.169	0.06	0.925	67	cm
BS = a*TL + B (sub)	0.196	−7.98	0.851	47	cm
BS = a*TL + B (adult)	0.204	−12.72	0.932	17	cm
G = a*TL + B	0.619	−15.21	0.958	163	cm

Frontal Span (FS), Lateral Span (LS), Proximal Span (PS), average Body Span (BS), and Total Length (TL). BS-TL relationships were analyzed independently for females (F) and males (M) as well as juveniles (juv), subadults (sub), and adults (adult). Girth (G) is derived from the circular approximation at the FS position.

**Fig 4:**
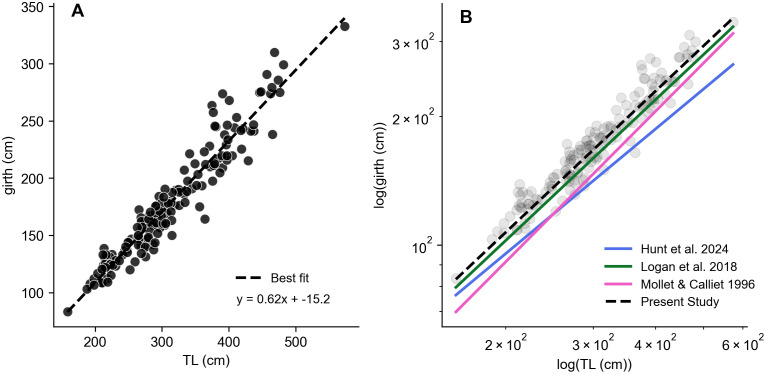
Girth-length scaling relationships across regional populations. **(A)** Scaling relationship between UAS-based white shark girth and total length (TL). Girth estimates were derived from the frontal span (40% TL body position) in drone estimates, converted to circular girth for comparison. **(B)** UAS-based estimates are compared to published scaling relationships for juvenile eastern Pacific (Logan et al. 2018), Australian (Hunt et al. 2024), and global (Mollet & Calliet 1996) white sharks measured post-mortem.

Converting LS to circular girth (G), the relationship aligned with previously published girth-length scaling relationships measured directly from animals post-mortem (Fig 4). The girth-length relationship reported in this study is the highest estimate of published relationships in white sharks, aligning most closely with observations of juvenile eastern Pacific white sharks (TL < 268 cm) [7]. The relationship deviated from Australian white sharks at larger body sizes, where white sharks reached overall lower girths even at large TLs [15]. However, girth values were more aligned at large body sizes for the generalized estimate across populations but deviated at small body sizes [14].

(c) **Body condition**

Shark body condition metric BSR demonstrated strong relationships with previously published condition metrics. BSR values were centered around 0.17 (mean:0.170 ± 0.011) and ranged from 0.138 to 0.210, while SCA values ranged from 0.0.83-1.27 (mean:1.02 ± 0.073), and computed BAI values ranged from 11-18 (mean:13.50 ± 1.05). Comparing BSR to these other metrics of relative body condition, we found close correlation with both BAI (R^2^ = 0.87, p < 0.001) and SCA (R^2^ = 0.99, p < 0.001).

**Fig 5:**
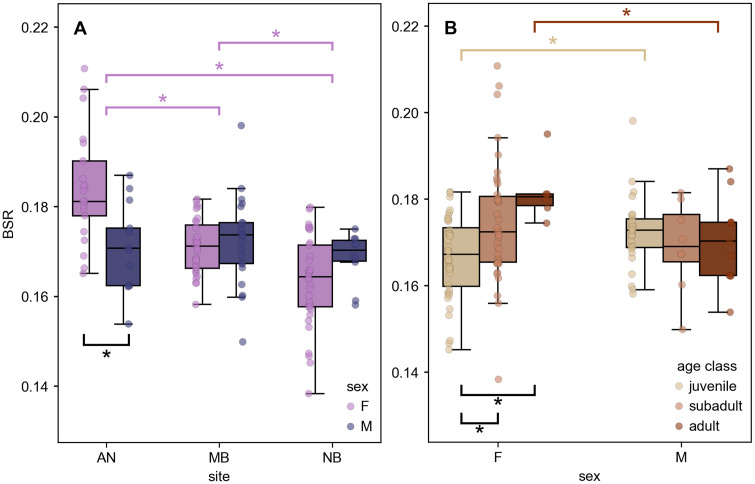
Body condition variation across ontogeny and sex. Body Span Ratio (BSR), or girth-length ratio, distributions demonstrate differences across demographic groups. **(A)** BSR by site demonstrates differences amongst females, as females show significantly higher condition than males at AN (sample size (n) by group: AN-F:21, AN-M:13, MB-F:31, MB-M:23, NB-F:33, NB-M:10). Site acronyms represent Año Nuevo (AN), New Brighton (NB), and Marina (MB). **(B)** BSR values differ across ontogenetic stages, or age class, amongst females but not males (sample size (n) by group: juv-F:40, juv-M:27, subadult-F:39, subadult-M:8, adult-F:6, adult-M:11). Significance (p < 0.05) indicated by (*).

Body condition, approximated by BSR, demonstrated site and sex-specific differences (Fig 5). Assumptions of normality and homogeneity of variance were met both across sites and within site-specific sex groups (all p > 0.05). Females demonstrated a positive relationship (r = 0.387, p < 0.001) between BSR and TL, indicating that larger females demonstrated higher body condition. However, males demonstrated no such relationship (r = −0148, p = 0.327), indicating that condition was relatively stable with TL. Due to the differences in condition scaling, we sex-stratified all further analyses to prevent confounding findings with sex ratios. Analyzing BSR across sites, we observed strong differences across sites for females (ANOVA, p < 0.001) but not males (ANOVA, p > 0.05). Among females, Tukey post-hoc tests revealed a progressive increase in BSR from NB to MB to AN. Within-site sex differences in BSR were significant only at AN, where females demonstrated higher BSR than their male counterparts (t = 3.120, p < 0.01). No significant sex differences in BSR were found at MB (t = −0.674, p = 0.503) or NB (t = −1.570, p = 0.124). BSR across age classes demonstrated that females had significantly different BSR across age classes (ANOVA with post-hoc Tukey, p < 0.01), while no significant differences were observed among male age classes. Juveniles and adults demonstrated significantly different BSR across sex, with males demonstrating higher BSR at the juvenile age class (p < 0.01) and females demonstrating higher BSR at the adult age class (p < 0.05).

## Discussion

Eastern Pacific white sharks exhibit distinct patterns of ontogenetic habitat use, with nearshore regions appearing to function as a continuum of life-stage specific habitats rather than discrete juvenile or adult zones. Establishing an efficient pipeline for extracting field-based measurements, this study provides robust morphometric data across aggregations and life stages. Comparisons across these data illuminate size-structured habitat partitioning among nearby white shark aggregations and highlight demographic and population-specific body condition. By situating morphological variation within population and aggregation-level contexts, this study provides new insights into how morphology shapes ecology in a top predator.

### Ecological application

(a) **Spatial habitat partitioning**

Rather than a discrete shift from nursery to adult habitats, white sharks appear to use a continuum of coastal habitats across ontogeny, with regions within Monterey Bay potentially serving as a key transitional zone. In elasmobranch ecology, juveniles often occupy distinct nursery habitats before migrating to riskier areas at greater body sizes and maturation [61]. While white shark nursery habitats in the eastern Pacific have been identified in warmer waters south of Point Conception, in the Southern California Bight, and the Baja peninsula [23,62], Monterey Bay has emerged in recent years as a hotspot for juvenile white sharks [27,28,39], though its local aggregations remain understudied. The discrete size compositions of aggregations within Monterey Bay and proximity to local adult sites suggest that this may act as a transition area as animals grow.

Electronic tagging data have also indicated a shift in habitat use across age classes along the coast of California. For example, juvenile white sharks tagged in Southern California with acoustic transmitters and short-term deployments of pop-up satellite archival tags have demonstrated initial evidence of an increasingly northward presence at older age classes [22,62]. Additionally, acoustic tagging studies from Año Nuevo have revealed that adults display higher residency to known aggregations while subadults exhibit broader coastal movements [49]. Future work may leverage acoustic telemetry and longer satellite tracking studies to confirm Monterey Bay’s role as a transition zone in the broader ontogenetic habitat continuum.

This study’s observation of spatial habitat partitioning along a thermal gradient within the MBNMS (Fig 2) suggests a more complex space-use paradigm influenced by both extrinsic and intrinsic factors. The largest sharks are predominantly found in the most northern and coldest site (11–13°C), while smaller individuals, which may have lower thermogenic capacity coupled with high surface area to volume ratios that increase heat loss in cooler water [22,63], occupy warmer sites in the MBNMS. However, even within Monterey Bay, clear size partitioning emerges, as NB, the warmest site during sampling (18–19°C), hosts significantly smaller individuals (primarily juveniles) than MB (16–17°C), which supports larger juveniles and small subadults. This pattern is consistent with evidence that juvenile white sharks are sensitive to small temperature fluctuations [64] and that juvenile thermal optima typically occur at higher temperatures than those of adults [65]. Consistent with these results, electronic tagging studies of white sharks along the California coast have also revealed preferences for water temperatures 16–19°C for juvenile white sharks [22,23,27] and 12–14°C for adults in nearshore habitats [66]. However, fine-scale thermal preferences across body size have not been resolved and juveniles across a range of shark species have also been shown to tolerate conditions outside their thermal optimum for various lengths of time [65]. Additional ecological pressures may also be influencing habitat selection within MBNMS, as nearshore habitats can provide protection from predation or competition [65]. The observed habitat partitioning may also be a result of avoidance of larger conspecifics [67]. In addition, foraging may be of importance for the size partitioning as white sharks feed on different target prey species across ontogeny [12,21]. However, sites within Monterey Bay (New Brighton, Marina) offer similar prey landscapes, rich in smaller fish and rays but lacking marine mammal aggregations. The two areas have similar coastal habitat structure, but demonstrate differing size compositions of shark aggregations, suggesting that temperature likely plays a role in shaping the observed partitioning with the smallest sharks in the warmest, most protected area. Given that juvenile white sharks have shown habitat shifts in response to interannual periods of ocean warming [27,68], future work disentangling thermal physiology, ecological interactions, and prey landscapes will be crucial to predicting changes under shifting climate regimes.

(b) **Morphometric relationships**

Whole-body morphometrics reflect key ecological and functional traits including energy stores, reproductive status, and hydrodynamic function. Our results confirm that body widths captured at standardized biometric markers (Fig 4) encompass the region of largest overall girth and possible body condition signal, consistent with prior studies of large predatory sharks [8,52]. While most body regions showed limited variation in proportional span (span-length ratio), the mid-body region exhibited the most variability, reflecting functional significance as a region influenced by energetic reserves or distension from pregnancy. This morphometric framework could be adapted to capture additional morphometrics traits, enabling a better understanding of hydrodynamics and swimming performance across ontogeny. For example, it has been noted that pectoral fin area scales negatively with body size, with relatively larger fins generating increased lift amongst smaller individuals to offset low buoyancy [15]. Adapting segmentation approaches to capture such traits that scale allometrically could also provide scale-independent metrics of body size directly from aerial imagery as UAS-based observations of sharks become more common, reducing reliance on rigorous photogrammetric calibration techniques. Additionally, segmentation analyses could be paired with body pose estimation to extract key anatomical landmarks, like dorsal fins and the caudal peduncle, opening avenues for exploring detailed functional analyses of free-ranging sharks.

Demographic differences in span-length scaling relationships within the eastern Pacific white shark population offers a window into how life-history and energetic demands diverge between sexes. The scaling relationships reported here extend existing findings, which have previously relied on direct measurements of immature white sharks [7], by providing expanded measurement flexibility that encompasses subadults and adults. Morphometrics across these age classes have yet to be fully evaluated, owing to the challenges of directly sampling large-bodied adults and limited fishery interactions in the protected MBNMS and Greater Farallones National Marine Sanctuaries. Additionally, adults are rarely observed post-mortem, contributing to a general sparsity of girth data. The results demonstrate sex-based differences in span-length scaling, whereby females demonstrate a higher rate of girth acquisition per unit length than males (Table 2), and related differences across ontogeny only amongst females. While previous work finds that girth-length relationships do not differ by sex, but can differ by age class, these studies acknowledge a restricted assessment of morphological data with samples dominated by immature individuals [7,15]. Authors note that with a larger sample of adults, sex differences may become detectable given that morphological size differences become more pronounced in the subadult and adult age class [14,15]. By including this broader size range in our study, we were able to resolve this limitation and detect significant sex-based differences in scaling relationships in the eastern Pacific population of white sharks.

Observed population-specific girth-length scaling may reflect regional differences in evolutionary morphology or prey availability. While the generalized girth-length relationship found in this study was elevated compared to Australian and global trends, close alignment with juvenile eastern Pacific estimates [7] reinforces regional relationships and reveals a robust overall trend across ontogeny. These findings are consistent with previous suggestions that eastern Pacific white sharks may acquire greater mass per unit length potentially owing to higher body condition [7,15] and substantial availability of prey in the California Current. Although previous studies suggested this to be a possible artifact of extrapolating from smaller size classes, we confirm this relationship exists across a broader size range. White shark life history characteristics have been shown to differ globally, with individual populations reflecting variation in growth rates, size-at-maturity, and terminal body sizes [9,18]. Genomic studies have recently clarified genetic differentiation amongst white shark populations by ocean basin [69,70], but phenotypic differences in morphology parameters like girth remain sparsely documented. As girth impacts body mass, both these regionally-specific and the previously-described sex-dependent relationships have implications for length-derived mass estimations used to compute mass-specific metabolic rates [71], feeding requirements [72] and energy budgets [73].

(c) **Body condition**

The alignment of the body condition ratio reported here with published indices supports the BSR as an appropriate and practical proxy for assessing body condition in white sharks. BSR demonstrated strong alignment with both BAI, a UAS imagery-derived index developed for large whales, and SCA, a metric based on direct measurements of other large shark species. Converted to BAI, values in this study were expectedly smaller (range: 11−18) than those reported for humpback and grey whales (range: 20−35) [32,41]. Approximated SCA values (range: 0.83-1.27) closely matched those reported for tiger sharks (range: 0.97-1.25) [52]. Observed BSR values for white sharks in this study (range: 0.138-0.210) also fell within the range of those reported for a singular captive juvenile white shark from the same population (range: 0.15-0.20) [6]. Moreover, as compared to other metrics, BSR provides advantages as a non-invasive alternative to direct measurement approaches [9] that can cause physiological stress [74] or logistical concerns at large body sizes. Additionally, as an average span-length ratio, this metric benefits from straightforward interpretability for comparison across taxa.

The emergence of sex-based differences in body condition at the adult life stage suggests that energetic status is modulated by physiological or ecological factors associated with maturity. While adult female white sharks exhibited higher body condition than adult males, juveniles and subadults showed no such sex-based differences (Fig 5B). Analyses of body condition within sex support this trend, with males demonstrating stable body condition across age classes and females gaining progressively higher body condition from juvenile to subadult to adult (Fig 5B). Previous studies of girth differences across age classes have had mixed results, with some studies finding significant differences amongst girth-length relationships [15] and others noting a constant girth-length ratio [14]. Given the observation of age class differences in condition only amongst females, it is possible that previous relationships were confounded by mixed-sex analyses. Sex-based differences in body condition have been found in tiger sharks and bull sharks [52], but were not found to be significant in previously published relationships for white sharks [7,15]. The increased body condition amongst adult females aligns with the transition to sexual maturity, whereby females may incur energetic requirements of gestation [75] or demonstrate swollen abdomens from pregnancy. While this relationship has not been well established in published literature, it has been noted that Atlantic female white sharks are recognized for maturity in part by high length-girth ratios [9,76].

Site-based differences in body condition among female white sharks, but not males, suggests that sex-specific reproductive or foraging strategies drive body condition rather than prey landscapes alone. Female white sharks demonstrated significant site-based differences in body condition, with highest condition amongst females at Año Nuevo, whereby their male counterparts displayed stable condition across aggregations (Fig 5A). As these sites are characterized by different shark size regimes (Fig 2B), this could be related to previously discussed age-based differences in condition reflected in a positive correlation between body condition and TL for females. Alternatively, site-based body condition differences could emerge from differing foraging strategies [13]. Sharks aggregating at Año Nuevo forage on calorically-dense marine mammals, including Northern elephant seals (*Mirounga angustirostris*), California sea lions (*Zalophus californianus*), and harbor seals (*Phoca vitulina*) [77,78], and build fat stores before making offshore migrations [6]. Juvenile sharks occupying protected coastal habitats like Marina and New Brighton are thought to feed on teleosts and other benthic prey throughout the year [22,23]. Although prey landscapes alone do not consistently explain site-specific differences body condition across both sexes, males and females may exhibit distinct prey preferences that influence body condition. Site-specific foraging patterns might also be expected to produce more variability in body condition at Año Nuevo given the possible “boom and bust” nature of migrating adults as they deplete body condition while transiting from offshore habitats [6]. While high variability in body condition amongst individuals at Año Nuevo was not apparent in this study, this may be confounded by differing arrival and departure times that have been noted across groups [49]. Additionally, females may exhibit various body condition signals related to their respective phase of gestation, which is anticipated to be up to 18 months long [79]. Future work with higher sampling density of repeated individuals in a given season could explore these foraging-related effects on body condition.

### Model development and validation

This study demonstrates that drone-based aerial photogrammetry paired with segmentation analysis offers an efficient, scalable approach to obtaining detailed morphometric information of large-bodied, free-swimming elasmobranchs. By enabling remote measurements of the study organism, this approach minimizes disturbances and expands the sampling power across individuals [80]. Additionally, sampling individuals *in-situ* preserves their natural body shape and avoids gravitational distortions during landings, while allowing for more comprehensive morphological analyses than would be possible during time-limited capture events. Moreover, this study expands upon previous work to pair drone data with underwater footage to acquire sex and identify unique individuals through fin-ID. Advances in the photo-identification of individuals via stable biometric markers [81], like dorsal fin morphology [82–84], spot patterns [85,86], or pigmentation [87,88], extend the applicability of this framework to a range of elasmobranch species. This novel approach enables a comparative analysis of measurements and body condition among demographic groups and the future ability to track individual shark morphometrics over time.

The segmentation model and post-processing workflow together achieved high performance in extracting shark body morphometrics from aerial imagery, despite being trained on a modest dataset (n = 888 images). This underscores the potential of deep learning in ecological applications, where limited training data are common but can be mitigated through evolving transfer learning and pre-training approaches [89]. Using a U-Net pre-trained on image database ImageNet, our highest-performing model configurations incorporated a custom morphometric procedure that conservatively cropped to the maximum possible shark body length. For increased flexibility, future refinements could integrate a prior object detection step to localize a bounding box around the animal (e.g., Gray et al., 2019) to guide cropping procedures. Importantly, high test set performance demonstrates the model’s generalizability to new individuals, conditions, and field days beyond this study. In this way, this study establishes a reliable method to extract shark morphometrics from aerial imagery without key points or physical markers, advancing non-invasive measurement approaches widely applied in marine mammals [90,91].

### Error analysis

Quantifying error in drone-based morphometrics using calibration objects revealed barometric variability that could introduce substantial measurement uncertainty. We mitigated this variation through the development of a custom correction protocol (S1 Fig). Low altitude error within a drone flight provided support for correcting altitude readings on a per-flight basis rather than a global fixed offset. Using calibration objects to correct GSD on a per-flight basis [57,92] or quantify measurement error [93] has been previously demonstrated through UAS-photogrammetry of marine mammals. While previous shark studies have utilized UAS for approximate measurements of TL [94], photogrammetric methods calibration have not always been reported. This photogrammetric approach generally follows the best practices put forth by Burnett et al. 2019 and Segre 2025, with the addition of a depth offset necessary for subsurface megafauna [45,95]. Propagating error estimations into our white shark dataset, we observed high uncertainty in low altitude imagery consistent with previous studies of UAS photogrammetry in marine megafauna [57,92]. This results from low altitude imaging inflating the impact of barometric error on GSD estimation. On the other hand, high altitude imaging reduces object resolution and subsequently increases the magnitude of digitization error with a larger GSD. To balance these trade-offs, we recommend imaging at intermediate altitudes (~20-60m) when using similar protocols. Applying an altitude threshold (>10m) and flight-based altitude calibration in our dataset yielded low expected measurement error (±3.90% TL), reinforced by consistent measurements when re-observing the same individuals over multiple images and flights (4.82%CV). Low uncertainty estimations as well as consistent measurements of individual white sharks across varied flight conditions supports the reliability of our measurement approach.

Benchmarking visual estimations with UAS-based measurements enables quantification of error in historical methods in ways previously not possible. While we observed consistent underestimations in visual estimations (~30 cm), drone and visual measurements were strongly correlated (r = 0.8), suggesting that prior size-class assignments and conclusion regarding age structure in this population [5,96] likely remain largely valid. Given that visual estimates were known to have variability [31], previous studies in this population have conservatively grouped individuals into broad age classes (e.g., [49]) rather than using continuous body size. By incorporating UAS-based measurements, or calibrating visual estimates with UAS data, future studies can move beyond coarse ontogenetic groupings, refining size and age class assignments while enabling ecological analyses across a continuum of body size.

## Conclusions

Ontogenetic variation in body size and morphology shapes ecological roles and animal physiology, yet capturing these traits and linkages in large-bodied predators has remained challenging. By combining high-resolution measurements paired with demographic context, this study enables connecting individual morphology to population-level ecological inference and life-history strategies. Morphometric analyses reveal distinct ontogenetic and sex-specific trends that capture shifts in habitat use, growth trajectories, and energetic stores, highlighting the varying ecological impacts of body size. Moreover, this framework establishes a generalizable approach for efficiently quantifying morphology and body condition in free-ranging, large-bodied species where direct measurements are challenging to obtain. The co-occurrence of juvenile, subadult, and adult white sharks within the MBNMS provided a uniquely productive natural system, enabling standardized morphometric comparisons across ontogeny that revealed size-structured habitat partitioning and ontogenetic shifts in body condition that would be difficult to resolve across spatially separated populations.

## Supporting information

S1 Fig Barometric altitude error and effect on shark Total Length (TL) measurements. (A) Distribution of across-flight altitude offsets, where each sample represents the flight-averaged difference between the barometric altitude and the ‘true’ altitude derived from calibration imagery (n = 236). (B) Distribution of within-flight altitude offsets, ‘corrected’ by subtracting the per-flight mean altitude offset and computing the per-image difference between the corrected barometric altitude and true altitude (n = 236). (C) Error introduced to shark Total Length (TL) by effective height (H), the combined error in drone barometer and shark swimming depth, across altitudes. Error decreases hyperbolically with increasing altitude, with a defined elbow at approximately 2.3m altitude. A conservative threshold of 10m was used to preserve high sample size while ensuring low error in the data.
(TIF)

S2 Fig Data distribution comparisons for day-individuals and biological individuals. Comparison of morphometric parameters (A) Total Length (TL) and (B) Body Span Ratio (BSR) averaged over day-individuals (n = 163) and biological individuals (n = 134). Alignment of the corresponding distributions indicate minimal effect of re-sampling day-individuals in this study. Biological individuals were all observed within the same site except for three individuals observed at both New Brighton and Ano Nuevo (n = 1) and New Brighton and Marina (n = 2).
(TIF)

S3 Fig Comparisons of girth-length scaling relationships across sex and ontogeny. Girth-length scaling relationships separated by (A) sex and (B) age class, with line of best fit equation displayed for each demographic subgroup. Girth is derived from the frontal span (40th body percentile) in drone estimates and converted to circular girth for comparison.
(TIF)
