# Supplementary figures and images for: Ontogenetic shifts in morphology and ecology of eastern Pacific white sharks revealed by computer vision
Source: PLoS One. 2026 May 20;21(5):e0348174. doi: 10.1371/journal.pone.0348174 (PMC13189333; doi:10.1371/journal.pone.0348174)

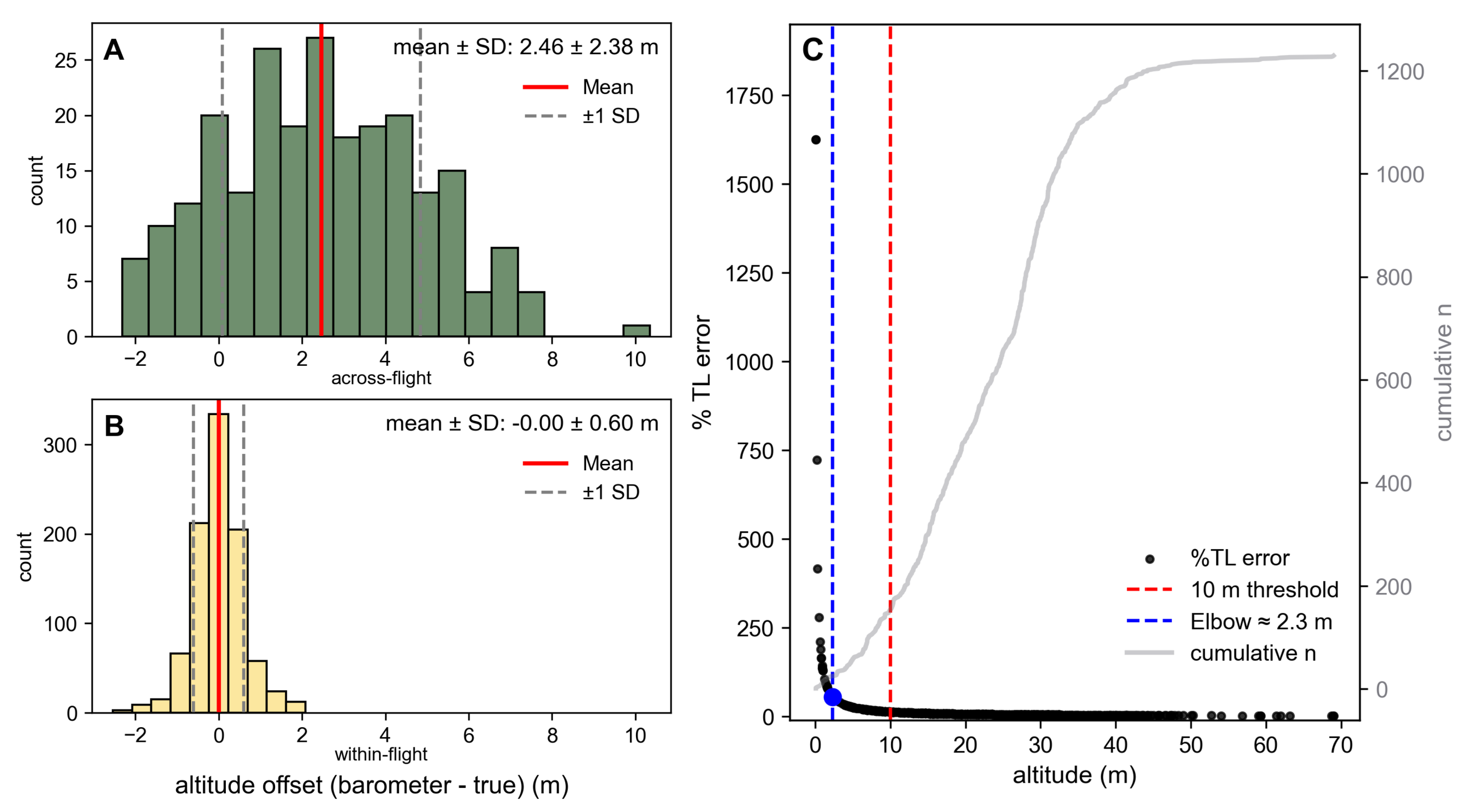

Supplement: S1 Fig — (A) Distribution of across-flight altitude offsets, where each sample represents the flight-averaged difference between the barometric altitude and the ‘true’ altitude derived from calibration imagery (n = 236). (B) Distribution of within-flight altitude offsets, ‘corrected’ by subtracting the per-flight mean altitude offset and computing the per-image difference between the corrected barometric altitude and true altitude (n = 236). (C) Error introduced to shark Total Length (TL) by effective height (H), the combined error in drone barometer and shark swimming depth, across altitudes. Error decreases hyperbolically with increasing altitude, with a defined elbow at approximately 2.3m altitude. A conservative threshold of 10m was used to preserve high sample size while ensuring low error in the data. (TIF) [file pone.0348174.s001.tif]

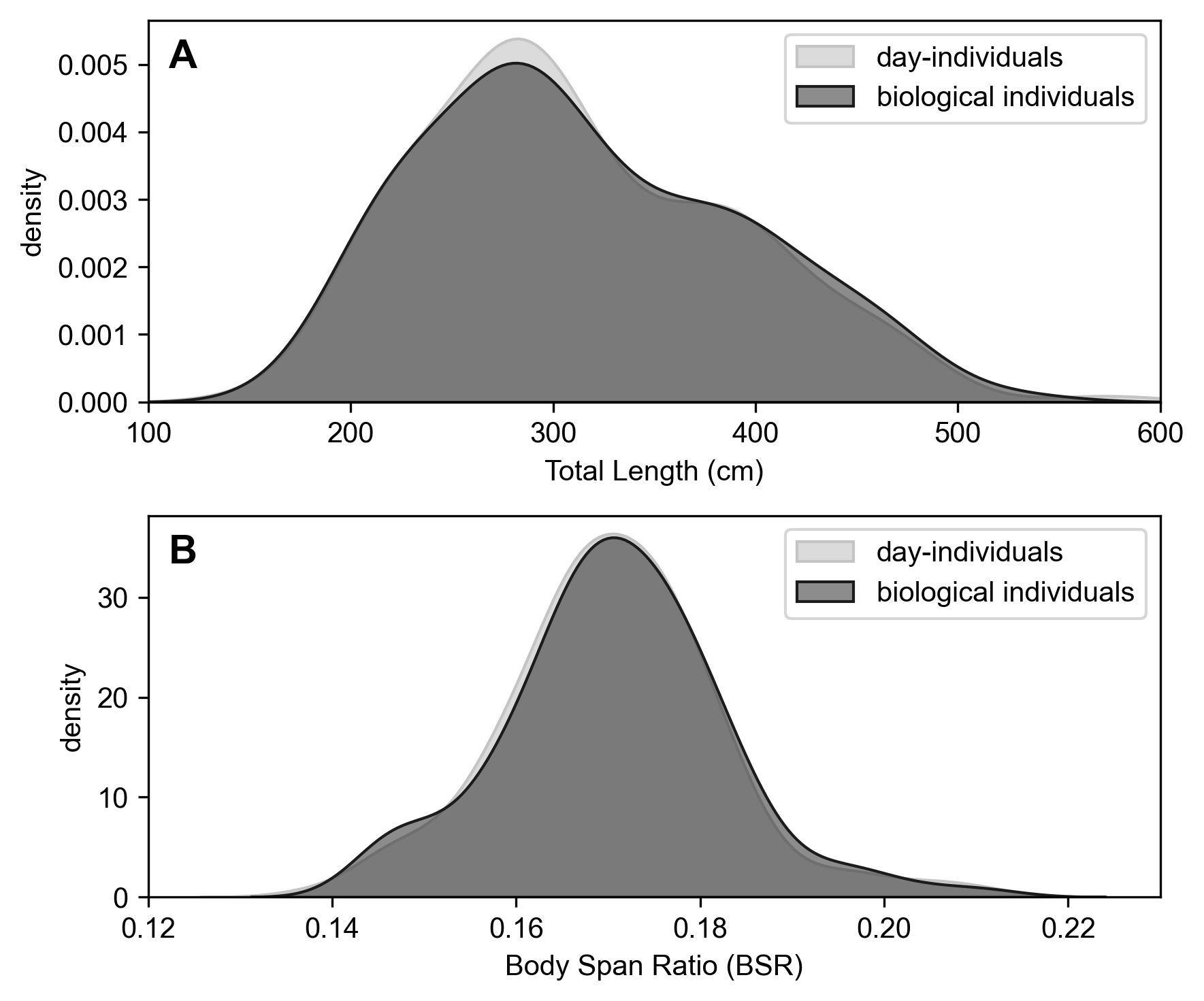

Supplement: S2 Fig — Comparison of morphometric parameters (A) Total Length (TL) and (B) Body Span Ratio (BSR) averaged over day-individuals (n = 163) and biological individuals (n = 134). Alignment of the corresponding distributions indicate minimal effect of re-sampling day-individuals in this study. Biological individuals were all observed within the same site except for three individuals observed at both New Brighton and Ano Nuevo (n = 1) and New Brighton and Marina (n = 2). (TIF) [file pone.0348174.s002.tif]

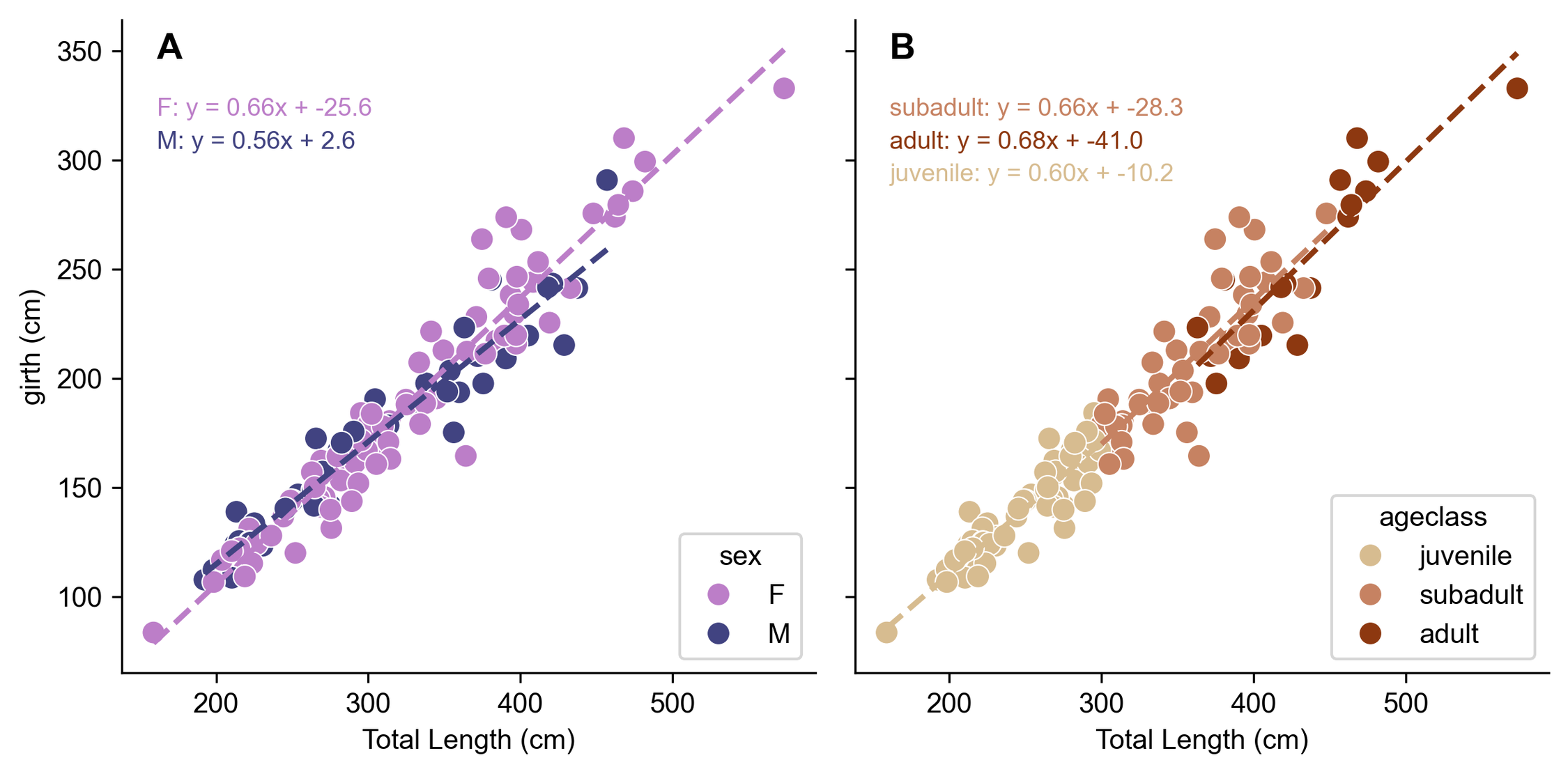

Supplement: S3 Fig — Girth-length scaling relationships separated by (A) sex and (B) age class, with line of best fit equation displayed for each demographic subgroup. Girth is derived from the frontal span (40th body percentile) in drone estimates and converted to circular girth for comparison. (TIF) [file pone.0348174.s003.tif]
